# Exposure of *Caenorhabditis elegans* to Dietary *Nε*-Carboxymethyllysine Emphasizes Endocytosis as a New Route for Intestinal Absorption of Advanced Glycation End Products

**DOI:** 10.3390/nu13124398

**Published:** 2021-12-08

**Authors:** Constance Dubois, Rachel Litke, Stéphane Rianha, Charles Paul-Constant, Jean-Marc Lo Guidice, Solenne Taront, Frédéric J. Tessier, Eric Boulanger, Chantal Fradin

**Affiliations:** 1 Univ. Lille, Inserm, CHU Lille, Institut Pasteur de Lille, U1167-RID-AGE-Facteurs de Risque et Déterminants Moléculaires des Maladies Liées au Vieillissement, F-59000 Lille, France; constance.dubois@univ-lille.fr (C.D.); rachel.litke@mssm.edu (R.L.); st.rianha@gmail.com (S.R.); charles.paul-constant@univ-lille.fr (C.P.-C.); solenne.taront@univ-lille.fr (S.T.); frederic.tessier@univ-lille.fr (F.J.T.); eric.boulanger@univ-lille.fr (E.B.); 2 Univ. Lille, CHU Lille, Institut Pasteur de Lille, ULR 4483-IMPECS-IMPact de l’Environnement Chimique sur la Santé Humaine, F-59000 Lille, France; jean-marc.lo-guidice@univ-lille.fr

**Keywords:** advanced glycation end products (AGEs), *Nε*-carboxymethyllysine (CML), *Caenorhabditis elegans*, intestine, absorption, enterocyte, endocytosis, lysosome, digestion

## Abstract

The impact of dietary advanced glycation end products (dAGEs) on human health has been discussed in many studies but, to date, no consensual pathophysiological process has been demonstrated. The intestinal absorption pathways which have so far been described for dAGEs, the passive diffusion of free AGE adducts and transport of glycated di-tripeptides by the peptide transporter 1 (PEPT-1), are not compatible with certain pathophysiological processes described. To get new insight into the intestinal absorption pathways and the pathophysiological mechanisms of dAGEs, we initiated an in vivo study with a so-called simple animal model with a complete digestive tract, *Caenorhabditis elegans*. Dietary bacteria were chemically modified with glyoxylic acid to mainly produce *Nε*-carboxymethyllysine (CML) and used to feed the worms. We performed different immunotechniques using an anti-CML antibody for the relative quantification of ingested CML and localization of this AGE in the worms’ intestine. The relative expression of genes encoding different biological processes such as response to stresses and intestinal digestion were determined. The physiological development of the worms was verified. All the results were compared with those obtained with the control bacteria. The results revealed a new route for the intestinal absorption of dietary CML (dCML), endocytosis, which could be mediated by scavenger receptors. The exposure of worms to dCML induced a reproductive defect and a transcriptional response reflecting oxidative, carbonyl and protein folding stresses. These data, in particular the demonstration of endocytosis of dCML by enterocytes, open up new perspectives to better characterize the pathophysiological mechanisms of dAGEs.

## 1. Introduction

Dietary advanced glycation end products (dAGEs) are formed during cooking as well as subsequent food processing, which can impact various factors such as the pH and the availability of water and transition metals, promoting glycation [1]. Given the number of alimentary products containing AGEs, many reports have rightly questioned the impact of dAGEs on human health [2,3]. Several observational and interventional studies, including parallel and crossover trials for the latter, were thus carried out.

In short, dAGEs would be aggravating factors in older adults and in people with certain pathologies, in particular metabolic and hormonal disorders and chronic kidney diseases [4,5,6,7,8,9,10,11]. In these populations, a low-AGE diet can decrease plasma and urinary AGEs levels together with inflammatory, oxidative stress and metabolic (e.g., insulin resistance, cholesterol, …) markers. However, studies have shown that not all, if any, markers are improved by dAGE restriction [3,10,12]. In healthy subjects, although some studies have shown the opposite [13], dAGEs have less impact on metabolic homeostasis and inflammation [14]; some diets rich in dAGEs did not even increase the levels of these products in plasma and urine [15,16]. The dAGEs would be effectively eliminated in those people.

Clinical studies have several limitations which do not allow a flawless interpretation. The size of the samples for some clinical studies is obviously a major limitation. Food monitoring of subjects is often at the discretion of the latter, making it difficult to control the diet actually followed. Different kinds of low/high-AGE diets were used in the various studies [3], allowing limited inter-study comparison. Diets low and high in AGEs often have other nutritional differences than the amount of dAGEs. The food processing necessary for the formation of AGEs, including heating, can alter essential nutrients for health [17]. A variety of AGEs are formed and it is unclear whether all of these products have the same complementary or adverse biological impacts [12,18,19]. In addition, the different analytical methods for assaying AGEs do not give consensual measurements for certain food matrices [18].

Some experimental models, mainly murine, of renal insufficiency or metabolic disorders have confirmed the induction of inflammation and metabolic complications by dAGEs [20,21,22]. The pathophysiological effects of dAGEs were also observed in healthy animals, where they promote oxidative and inflammatory processes and impair metabolic homeostasis [23]. However, as with clinical studies, the use of different diets from one experimental study to another complicates the inter-study comparisons. Glycation of foods has indeed been induced by various processes including heating [24], irradiation [25] or addition of dicarbonyl products [26]. All these processes can alter certain nutrients and can therefore bias the comparison of glycated vs. non-glycated diets. The precursors of AGEs, such as dicarbonyl products formed during food processing [27], can induce endogenous glycation, the pathophysiological impact of which can be falsely attributed to dAGEs.

In order to work with better defined dAGEs in terms of quantity and structure, various experimental studies have used protein supplements such as casein, soy protein, ovalbumin or bovine serum albumin (BSA), glycated vs. non-glycated, added to conventional animal food or given by oral gavage [28,29,30,31]. However, the choice of protein supplement is delicate and can lead to analytical bias. Indeed, many protein supplements, which are not part of the classic diet of animals have specific physiological properties [32,33]. The glycation of these supplements can alter their structural conformation and inhibit their biological activities, with a possible health impact that is independent of dAGE consumption.

The implication of dAGEs on health would depend on different mechanisms including their interaction with the receptor for advanced glycation end products (RAGE), which is certainly one of the most described [34]. Dietary or endogenous AGEs would thus have common pathophysiological mechanisms. Outside of the intestine, direct activation of the receptor by dAGEs seems unlikely because only free AGE adducts would pass the intestinal barrier, while only larger glycated peptides bind to and activate RAGE [35,36]. It cannot be ruled out that the accumulation of larger glycated peptides in the intestine, due, among other things, to the slower digestion of glycated proteins, could lead to their transcytosis [37]. Likewise, some chronic diseases and other conditions induce the rupture of tight junctions in the intestinal epithelium [38], leading to possible paracellular transport of these glycated peptides. To date, although the transport of glycated peptides through transcytosis or the paracellular absorption pathway is not excluded, no study has demonstrated it. Only the passive diffusion and absorption of glycated di-tripeptides, and more rarely free dAGEs, by the the peptide transporter 1 (PEPT-1) have been demonstrated [39,40]. Then the intracellular digestion of the di-tripeptides allows the release of dAGEs which can diffuse throughout the body [41].

The promotion of food allergies by ingestion of dAGEs is suggested in a growing number of reviews [42,43]. On the one hand, structural modification and incomplete digestion of glycated allergenic proteins could promote the formation of neo-allergens, which is a source of food allergy. On the other hand, although no study has yet shown it, RAGE, which is involved in pulmonary allergies via the induction of a T helper type 2 (TH2) response after interaction with certain alarmins [44], may be involved in the allergenic effects of AGEs. The AGE–RAGE axis, which seems to have a key role in the pathogenicity of endogenous and dAGEs, is regulated by various factors including other AGE receptors. Although being suspected of scavenging AGEs by disposing of them, these receptors, such as cluster of differentiation (CD) 36, also known as scavenger receptor class B member 2 (SR-B2) and one member of the AGE receptor complex (AGE-R), AGE-R1/ oligosaccharyl transferase 48 kDa subunit (OST-48), could be involved in the pathophysiology of dAGEs as for endogenous AGEs [45,46]. A recent experimental study has demonstrated an alternative pathophysiological mechanism of dAGEs, which promotes inflammation and kidney injury [47]. In this study, the heat-induced AGEs increased intestinal permeability and induced systemic innate immune complement activation.

Faced with these various promising but incomplete data on the fate of dAGEs in vivo and their impact on health, we aimed to use a so-called simple organism with a complete digestive tract, the nematode *Caenorhabditis elegans*, to initiate the evaluation of intestinal digestion and the uptake of dAGEs with the resulting physiological consequences. We exposed the worms to their classic laboratory food, *Escherichia coli* bacteria, glycated or non-glycated. For glycated food, bacteria were chemically modified with glyoxylic acid to mainly produce *Nε*-carboxymethyllysine (CML) [48]. We performed different immunotechniques using an anti-CML antibody for relative quantification of ingested CML and localization of this AGE in the worms’ intestine. The relative expression of genes encoding different biological processes such as response to different stresses and intestinal digestion were determined. The physiological development of the worms was verified. Our results highlighted a new route of intestinal absorption of dietary CML (dCML), endocytosis, which could be receptor-mediated. Exposure of worms to dCML induced a reproductive defect and a transcriptional response reflecting oxidative, carbonyl and protein folding stresses.

## 2. Materials and Methods

### 2.1. Nematode and Bacterial Strains

The Bristol N2 wild type strain and the OP50 *E. coli* feeding strain were obtained from the *Caenorhabditis* Genetics Center (https://cgc.umn.edu, accessed on 6 December 2021).

### 2.2. Culture of OP50 Strain and Maintenance of C. elegans

OP50 bacteria was grown in lysogeny broth medium (LB: tryptone 10 g/L, yeast extract 5 g/L and NaCl 10 g/L) at 37 °C with stirring. Worms were maintained at 20 °C on nematode growth medium (NGM: agar 20 g/L, peptone 2 g/L, NaCl 41 mM, KH_2_PO_4_ 20 mM pH 6.0, MgSO_4_ 1 mM, CaCl_2_ 1 mM and cholesterol 5 mg/L) agar plates seeded with OP50 bacteria. Before each experiment, eggs were harvested from gravid worms by lysis with sodium hypochlorite. Young adult worms were then obtained from synchronized L1 larvae after culture of eggs for 20 h in M9 buffer (NaCl 86 mM, Na_2_HPO_4_ 42 mM, KH_2_PO_4_ 22 mM and MgSO_4_ 1 mM).

Except for maintenance and larval development, feeding bacteria were 10 times concentrated and heat-inactivated 30 min at 65 °C.

### 2.3. Preparation of CML-Bound BSA and CML-Bound Bacteria

CML-bound BSA (BSA-CML) was prepared by reaction of glyoxylic acid with BSA, as described previously [29]. Briefly, BSA was incubated in 0.2 M phosphate buffer pH 7.8 containing 60 mM of glyoxylic acid and 180 mM of reducing agent, sodium cyanoborohydride. Heat-inactivated OP50 bacteria were glycated using the same method with either 20 mM or 60 mM of glyoxylic acid and 60 mM or 180 mM of sodium cyanoborohydride, respectively. Control BSA or heat-inactivated bacteria were incubated under the same conditions without glyoxylic acid. After 17 h of incubation at 37 °C, the 2 BSA preparations were dialyzed against ultrapure water and the bacteria were washed several times with M9 buffer. Glycation of BSA and bacteria was confirmed by western blot, and for bacteria only, by immunofluorescence with an anti-CML antibody (ab27684, Abcam, Paris, France).

### 2.4. Immunofluorescence of Control and CML-Bound Bacteria

Bacteria were fixed with buffered formaldehyde 4% pH 7.0 and washed in phosphate buffer saline (PBS: 0.01 M Na_2_HPO_4_, 0.0018 M KH_2_PO_4_ pH 7.4, 0.137 M NaCl and 0.0027 M KCl) prior to being immobilized on poly-L-lysine coated glass slide. The bacteria were then permeabilized with absolute ethanol. After blocking with 0.1% BSA in PBST (PBS containing 0.05% Tween), bacteria were incubated 2 h at room temperature with anti-CML antibody diluted 1:200 in PBST. After several washes, bacteria were incubated 2 h at room temperature with Alexa Fluor 568-conjugated antibody (anti-rabbit IgG, Molecular probes, Eugene, OR, USA) and Alexa Fluor 488-conjugated concanavalin A or Con A (Invitrogen, Villebon-sur-Yvette, France) diluted 1:1000 and 1:100 in PBST containing 2.5 g/L of 4′,6-diamidino-2-phenylindole (DAPI, Thermo Scientific, Courtaboeuf, France).

Slides were examined under a ZEISS Axiophot fluorescent microscope using Adobe Image Ready CS software (Adobe Systems France SAS, Paris, France).

### 2.5. Pepsin Digestion of Control and CML-Bound Bacteria and BSA

BSA and bacteria were resuspended in PBS at a protein concentration of 2 g/L. HCl was added to the BSA or bacteria solutions to a final concentration of 0.04 N. Pepsin (Sigma Aldrich Chimie SARL, Saint-Quentin-Fallavier, France) was added using enzyme:protein ratio of 1:20. After 8 h of incubation at 37 °C, reactions were stopped by heating at 95 °C for 10 min and analyzed by sodium dodecyl sulfate (SDS) polyacrylamide gel electrophoresis (PAGE) with 12% acrylamide as previously described [49,50] and western blot with anti-CML antibody as described later.

### 2.6. Exposure of Worms to dCML

For each condition, day 0 adult worms were grown several days at 20 °C in 25 cm^2^ tissue culture flask with vented cap (approximately 1000 worms/mL) containing 10 mL liquid synthetic medium (S medium: 50 mM KH_2_PO_4_ pH 6.0, 100 mM NaCl, 1 M potassium citrate pH 6.0, 3 mM MgSO_4_, 3 mM CaCl_2_, 1 mM ethylenediaminetetraacetic acid (EDTA) disodium, 1 mM FeSO_4_, 1 mM MnCl_2_, 1 mM ZnSO_4_, 1 mM CuSO_4_ and 5 mg/L cholesterol) supplemented with 50 μM 5-fluoro-2′-deoxyuridine (FUdR) to prevent the reproduction of the worms.

For experiments with protein supplement, S medium contained heat-inactivated bacteria and 1.6 mg/mL CML-bound BSA or control BSA.

For experiments with dietary bacteria, S medium was supplemented with CML modified bacteria, which were glycated with 20 or 60 mM glyoxylic acid (G20 and G60, respectively), or control bacteria (G0).

For some experiments, S medium was supplemented with both G0 and G60 bacteria at different G0:G60 ratio.

Before adding the worms to the different media, 50 μL of each medium was taken for quantification of CML by dot blot.

At different given times, 150 μL or 1 mL of worms were taken for each analysis.

To analyze the formation of progeny after exposure of the worms to dCML or to the control, after 24 h of culture, 200 μL of worms from each condition were taken and extensively washed with M9 buffer. Worms were resuspended in 2 mL of their respective media without FUdR and grown at 20 °C in 6-well plates. After 2 days of incubation, the number of eggs and larvae per worm was determined.

All the experiments were carried out in 3 biological replicates.

### 2.7. Whole Protein Extraction and Western and Dot Blotting

The soluble and insoluble protein fractions of the bacteria were obtained after grinding in the lysis buffer (100 mM Tris pH8.0, 140 mM NaCl, 20 mM EDTA and 1% SDS with protease inhibitors, Roche, Mannheim, Germany) with glass beads (<106 μm; Sigma Aldrich Chimie SARL, Saint-Quentin-Fallavier, France) using a homogenizing bead mill (Fisherbrand Beadmills, Fisher Scientific SAS, Illkirch, France) at 4 m/s, 3 times 10 s, 4 cycles. After centrifugation at 15,000 × *g* for 15 min at 4 °C, the supernatant containing the soluble fraction was separated from the insoluble pelleted fraction. The protein fractions (2 μg of each fraction) were analyzed by SDS-PAGE with 12% acrylamide as described before [49,50].

Total extracts of *C. elegans* were obtained by heating worms 10 min at 95 °C in lysis buffer (100 mM Tris pH 8.0, 140 mM NaCl, 20 mM EDTA and 5% SDS with protease inhibitors (Roche, Mannheim, Germany) and 10 μg of each extract were analyzed by SDS-PAGE with 10% acrylamide.

Membranes were probed with anti-CML and, for worms’ extracts, anti-actin (A2066, Merck, Sigma Aldrich Chimie SARL, Saint-Quentin-Fallavier, France) antibodies diluted 1:5000 and 1:2500, respectively, and then a horseradish peroxidase (HRP)-conjugated anti-rabbit IgG (Cell Signaling Technology, Danvers (MA), USA) diluted 1:5000. Enzyme activity was detected with Clarity western ECL substrate (BioRad, Marne-la-Coquette, France). The chemiluminescent signals were detected using the Fusion FX Spectra system (Vilber, Collégien, France).

For dot blotting of worms’ extracts, 0.2 μg of each worms’ protein extract was spotted in duplicate on nitrocellulose membranes. Membranes were probed and detected as described for western blotting. For each dot, CML signal intensity was measured with ImageJ software and was normalized to actin.

For the semi-quantification of CML in culture media, an equal volume of each medium was mixed with the worm’s lysis buffer. After 10 min of heating at 95 °C, 1 μL of each solution was spotted in duplicate on nitrocellulose membranes. Five BSA-CML standards with known increasing concentration of CML were used to estimate the concentration of CML in the culture media. Each standard was diluted in S medium, treated like the different culture media and similarly spotted. Membranes were stained with ponceau S before being probed with anti-CML antibody and detected as described above. For each dot, CML signal intensity was measured with ImageJ software, normalized to ponceau S staining and compared to the BSA-CML standards. The concentration of CML in the culture media was expressed in mM of CML equivalent from BSA-CML.

### 2.8. Isolation of Total RNAs and Gene Expression Analysis

At the different analysis times, the worms’ pellets were quickly frozen in liquid nitrogen. The worms were then homogenized in Trizol (Ambion, Huntingdon, UK) with acid-washed glass beads (425–600 μm; Sigma Aldrich Chimie SARL, Saint Quentin Fallavier, France) using a homogenizing bead mill at full speed (6 m/s), 3 times 10 s, 3 cycles. Total RNAs were extracted as previously described [51]. RNAs were treated with DNaseI (Thermo Scientific, Courtaboeuf, France) prior to being reverse transcribed to cDNA (High-capacity cDNA reverse transcription kits, Applied Biosystems, Villebon-sur-Yvette, France).

Quantitative real-time PCR was performed using PowerUp SYBR Green 2X Master Mix (Applied Biosystems, Villebon-sur-Yvette, France). Gene levels were normalized to 2 housekeeping genes that encode cell division cycle protein 42 (CDC-42) and peroxisomal membrane protein 3 (PMP-3) and relative expression was calculated by the 2^−ΔΔCt^ method. The relative expression of genes was analyzed by comparing the condition with dCML to the same condition without dCML. Primers are listed in Table S1.

### 2.9. Immunohistochemistry

The Bouin’s tube fixation protocol with slight modifications was followed [52]. Briefly, for each condition, approximately 150 worms were washed with M9 buffer and fixed in an equal volume of Bouin’s solution and methanol with 0.125 M dithiothreitol (DTT). Worms were then permeabilized by a rapid freeze-thaw cycle followed by repeated incubations in borate/triton buffer (0.01 M DTT, 1 M H_3_BO_3_, 0.5 M NaOH and 0.5% Triton X-100). After blocking with BSA 0.1% in PBS with 0.5% Triton X-100 and 1 mM EDTA disodium, worms were incubated overnight at room temperature with primary antibodies diluted in blocking solution: anti-CML 1:200, anti-IFB-2 (MH33, DSHB, Iowa city, IA, USA) 1:40, anti-RME-1 (RME1, DSHB) 1:10, anti-DYN-1 (DYN1, DSHB) 1:10 and anti-LMP-1 (LMP1, DSHB) 1:10. After several washes, worms were incubated 2 h at room temperature with secondary antibodies conjugated to either fluorescein isothiocyanate, FITC (anti-rabbit IgG, Molecular probes, Eugene, OR, USA) or Alexa Fluor 568 (anti-mouse IgG, Molecular probes) diluted respectively 1:200 and 1:1000 in blocking buffer containing 2.5 μg/μL of DAPI (Thermo Scientific, Courtaboeuf, France). For Con A staining, worms were incubated 2 h at room temperature with Alexa Fluor 488-labelled Con A diluted 1:100 in blocking buffer.

Slides were examined under a ZEISS spinning disk confocal microscope using ZEN 3.3 software (Zeiss, Marly-le-Roi, France).

### 2.10. Statistical Analyses

Statistical analyses were generated by bioinformatics software, GraphPad Prism version 8.0 (GraphPad Software, San Diego, CA, USA).

Gene expression analysis: all experiments were performed in 2 technical replicates and 3 biological replicates and the results were expressed as mean ± standard deviation (SD). Significant differences (*p* ≤ 0.05) between the means were determined by the Kruskal–Wallis, Dunn’s multiple comparisons test.

Dot blotting analysis: all experiments were performed in 3 biological replicates and the results were expressed as mean ± SD. Significant differences (*p* ≤ 0.05) between the means were determined by 2-way ANOVA, Tukey’s multiple comparisons test.

Progeny analysis: all experiments were performed in 3 biological replicates and the results were expressed as mean ± SD. Significant differences (*p* ≤ 0.05) between the means were determined by Mann–Whitney test.

## 3. Results

### 3.1. Glycation of Bacteria and Detection of CML Epitopes

Bacteria being the main food of the worms in the laboratory, we decided to feed *C. elegans* with this glycated or non-glycated food. In order to reduce the diversity of the AGEs formed and thus to facilitate the interpretation of the results, glyoxylic acid was chosen as the glycating agent. Treatment of proteins with glyoxylic acid mainly modifies lysine to CML, which is one of the most studied major endogenous and exogenous AGEs [48]. In adulthood, *C. elegans* can be grown with either living or dead bacteria. The use of dead bacteria limits the side effects linked to bacterial metabolism. Before glycating, the bacteria were heat inactivated for 30 min at 65 °C. Two concentrations of glyoxylic acid were used in order to obtain bacteria with different rates of CML.

**Figure 1:**
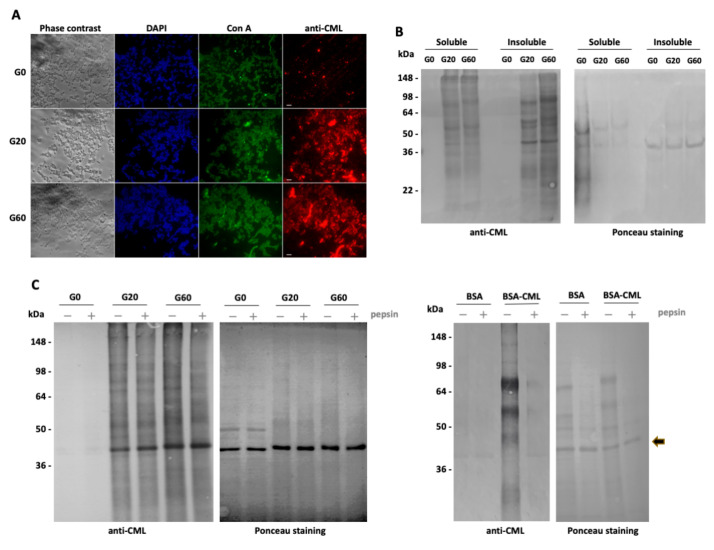
Glycation of bacteria and control of membrane and parietal integrities. (**A**) Indirect immunofluorescence assays were performed on bacteria, which were pretreated with 0 mM (G0), 20 mM (G20) or 60 mM (G60) of glyoxylic acid, using anti-*Nε-*carboxymethyllysine (CML) antibody and lectin concanavalin A (Con A). Bacteria DNA was stained with DAPI. For each type of bacteria, phase contrast and fluorescence micrographs with the mentioned DNA-binding probe, lectin or antibody are shown. Scale bars = 2 μm. (**B**) Western blot of soluble and insoluble protein extracts from G0, G20 or G60 bacteria was detected with anti-CML antibody. Before immunostaining, the membrane was stained with ponceau S for loading control. (**C**) Western blots of extracts from G0, G20 or G60 bacteria or non-glycated bovine serum albumin (BSA) or glycated BSA (BSA-CML) were stained with anti-CML antibody. Before electrophoresis, non-glycated and glycated bacteria or BSA were incubated without (−) or with (+) pepsin in acidic solution. Before immunostaining, the membranes were stained with ponceau S for loading control. The acidic condition induced the proteolysis of BSA with fragments of different sizes detectable after electrophoretic separation. One of these fragments, marked with an arrow, is known to be resistant to pepsin digestion [53]. All results are representative of 3 independent experiments.

We first confirmed the glycation of the bacteria by immunofluorescence with an anti-CML antibody. As expected, only bacteria treated with glyoxylic acid, regardless of its concentration, contained CML epitopes (Figure 1A). In contrast, all bacteria, glycated or non-glycated, were stained with Con A. This lectin is specific for α-D-mannose and α-D-glucose. In *E. coli*, it mainly recognizes exopolysaccharides. In order to have a better overview of the bacterial proteins that were glycated, we lysed the bacteria with a bead mill homogenizer and analyzed the soluble and insoluble fractions by western blot. Only the proteins from bacteria treated with glyoxylic acid had CML epitopes (Figure 1B). Several soluble and insoluble proteins were glycated, confirming homogeneous glycation of the bacteria with glyoxylic acid. As expected, more CML epitopes were detected on proteins from G60 bacteria, which were glycated with the highest amount of glyoxylic acid (60 mM).

### 3.2. Analysis of the Integrity of CML-Bound Bacteria

Before feeding the worms with the inactivated bacteria, glycated or not, we wanted to verify that the glycation of the bacteria did not lead to a strong loss of outer membrane and cell wall integrities. Immunofluorescence analyses, which were carried out with DAPI, showed that glycated and non-glycated bacteria had a similar staining of their DNA, excluding the possibility of leakage of the latter following glycation (Figure 1A). Additional analyses were performed to assess the susceptibility of bacteria to pepsin digestion. The outer membrane and the cell wall of the bacteria effectively prevent the passage of enzymes to digest proteins. Digestion of bacterial proteins by proteases seems unlikely without alteration of the cell wall and membranes of the bacteria, as when these are ground in the pharynx of *C. elegans*. Unlike BSA, glycated or not, used as a control, almost no bacterial protein was degraded by pepsin, whatever the glycation of the bacteria (Figure 1C). These results suggest that the integrity of heat-inactivated bacteria was maintained after glycation.

### 3.3. Glycated Bacterial Proteins Were Ingested by the Worms and Impaired Their Reproduction

**Figure 2:**
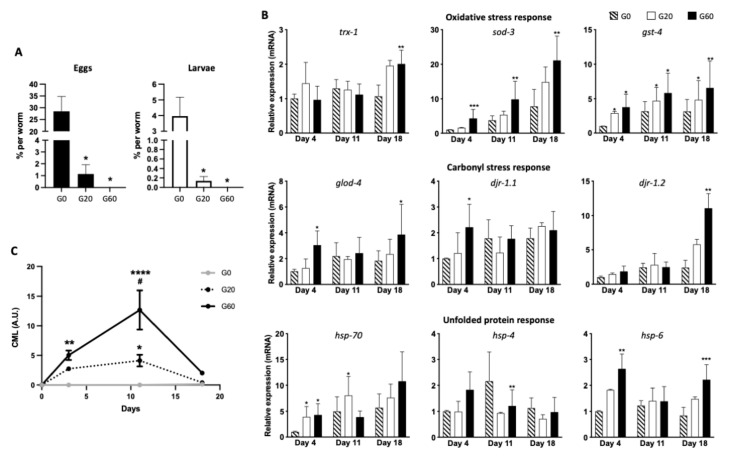
Effects of dietary *Nε*-carboxymethyllysine (dCML) exposure on worms’ progeny, transcriptional response to different stresses and levels of CML epitopes inside the worms. (**A**) Young adult worms were fed with bacteria, which were pretreated with 0 mM (G0), 20 mM (G20) or 60 mM (G60) of glyoxylic acid, in medium containing 5-fluoro-2′-deoxyuridine (FUdR). After 24 h of incubation, worms were grown for 2 days in the same conditions without FUdR. Number of eggs and larvae per worm present in the media were determined. Data were expressed as means ± standard deviation (SD) of 3 biological replicates. * *p* ≤ 0.05 G20 or G60 vs. G0 (Mann–Whitney test). (**B**) Young adult worms were fed for 4, 11 and 18 days with G0, G20 or G60 bacteria. Relative expression of gene coding for antioxidant, carbonyl stress and unfolded protein responses were analyzed by comparing all the conditions to the G0 at day 4. Data were expressed as means ± SD of three biological replicates. The *cdc-42* and *pmp-3* genes were used to normalize levels of gene expression. * *p* ≤ 0.05, ** *p* ≤0.01, *** *p* ≤ 0.001 G20 or G60 vs. G0 for the same incubation time (Kruskal–Wallis, Dunn’s multiple comparisons test). (**C**) Dot blots of lysates from worms fed 4, 11 and 18 days with G0, G20 or G60 bacteria were stained with anti-CML and anti-actin antibodies. CML and actin epitopes were quantified with ImageJ software and relative quantification of CML epitopes was normalized to actin. Data are expressed in arbitrary units (A.U.) and presented as the means ± SD of 3 biological replicates. * *p* ≤ 0.05, ** *p* ≤ 0.01, **** *p* ≤ 0.0001 G20 or G60 vs. G0, # *p* ≤ 0.05 G60 vs. G20 (2-way ANOVA, Tukey’s multiple comparisons test).

We exposed young adult worms to diets containing either non-glycated or glycated bacteria. Media with G20 and G60 contained 0.975 mM ± 0.053 and 1.252 mM ± 0.096 of CML equivalent, respectively. Before studying the ingestion and digestion of dCML by *C. elegans*, we ensured that our diets rich in CML did not induce toxicity in the worms. By maintaining the culture of the worms up to 18 days, we found that dCML had no drastic effect on the survival of the worms at least until this incubation time (data not shown). On the other hand, we noticed that the worms incubated with the 2 glycated diets contained fewer embryos. We therefore analyzed in more detail the formation of progeny after exposure of the worms to dCML or to the control. After 24 h of culture in the presence or absence of dCML, the worms were incubated for 2 days in their respective media without FUdR. The progeny, eggs and larvae, present in the media were counted. No offspring was found in the medium containing the most dCML, G60 (Figure 2A). Worms incubated in medium with less glycated bacteria (G20) produce 96% less eggs and larvae than those incubated in medium without CML. These results suggest that diets rich in CML have a significant effect on the reproduction of worms.

### 3.4. dCML Induced Different Stresses

We wondered if this reproductive defect could be due to a poor nutritional intake of glycated bacteria. We therefore analyzed the expression of the thioredoxin 1 encoding gene, *trx-1*, whose expression increases during nutritional restrictions, in particular by maintaining redox homeostasis [54]. After 4 and 11 days of culture of the worms, the expression of *trx-1* was not significantly modulated by dCML, suggesting that the worms did not undergo stress linked to a nutritional defect (Figure 2B). On the other hand, the key antioxidant genes of *C. elegans*, *sod-3* and *gst-4* coding respectively for mitochondrial superoxide dismutase 3 (SOD-3) and glutathione S-transferase 4 (GST-4), were significantly more expressed after 4, 11 and 18 days of incubation with dCML (Figure 2B). Significant induction of *sod-3* by dCML was only observed for the most glycated diet (G60), whereas the expression of *gst-4* was significantly induced by the 2 glycated diets. Higher expression of *sod-3* and *gst-4* in CML-rich diets suggests induction of an oxidative environment by these diets which may be associated with the reproductive defect of worms.

Oxidative environments can promote carbonyl stress with the production of potent glycating agents, the α-ketoaldehydes such as glyoxal and methylglyoxal [55]. Studies have indeed shown that diets rich in AGEs can induce the formation of endogenous AGEs [29]. We therefore analyzed the expression of genes encoding glyoxalases which convert α-ketoaldehydes into α-hydroxyacids [56]. *C. elegans* possesses 3 glyoxalases which are either dependent on glutathione, GLOD-4 (mammalian GLO1), or independent of this cofactor, DJR-1.1 and DJR-1.2 (mammalian DJ-1). Only the diet containing the most CML (G60) significantly induced the expression of *glod-4* and *djr-1.1* at day 4 (Figure 2B). This induction however seemed to be transient, since on day 11, no modulation of the expression of these genes was detected and on day 18, expression of *glod-4* and *djr-1.2* was induced by G60. After 18 days of culture, the worms were getting old and differences related to the aging of the worms may add to the effects of dCML. Moreover, at this time, the worms incubated with diets rich in CML expressed more *trx-1*, suggesting that dCML could have an effect on the worms’ aging, possibly by alteration of the redox homeostasis (Figure 2B).

As redox homeostasis is very important for proper protein folding which can be impaired by other sources of oxidative stress, we analyzed the expression of the genes coding for key chaperones of the unfolded protein response (UPR): the cytoplasmic heat shock proteins (HSP) 16.2 and 70, the mitochondrial HSP-6 and the endoplasmic reticulum (ER) HSP-4. The genes coding for HSP-70 and HSP-6 were upregulated in worms incubated 4 and 18 days with glycated bacteria (Figure 2B and Figure S1). Expression of HSP-16.2 encoding gene was significantly regulated at different incubation times; it increased at day 4 in the presence of dCML while it decreased at days 11 and 18 under the same conditions. These results suggest impairment of protein folding in the cytoplasm and mitochondria under glycating conditions, at least the most glycated condition (G60). Concerning *hsp-4*, its expression was less regulated than *hsp-70* and *hsp-6*. Glycated conditions reduced *hsp-4* expression at day 11.

### 3.5. Glycated Bacterial Proteins Are Digested by the Worms

In order to better understand the impact of dCML on the physiology of worms, we monitored this AGE over time. For this, we measured the relative expression of CML epitopes detected by dot blot. As expected, almost no CML epitope was detected in the worms’ lysates incubated in the control medium while the lysates of worms grown with the two glycated media contained detectable levels of CML (Figure 2C). More CML epitopes were measured in lysates of worms exposed to G60 than to G20. The amount of CML found in the worms increased over time before decreasing, with many fewer epitopes present at day 18. This detection profile is in line with 1—ingestion of glycated bacteria by the worms over time, 2—less ingestion of food by older worms and 3—digestion of glycated proteins.

**Figure 3:**
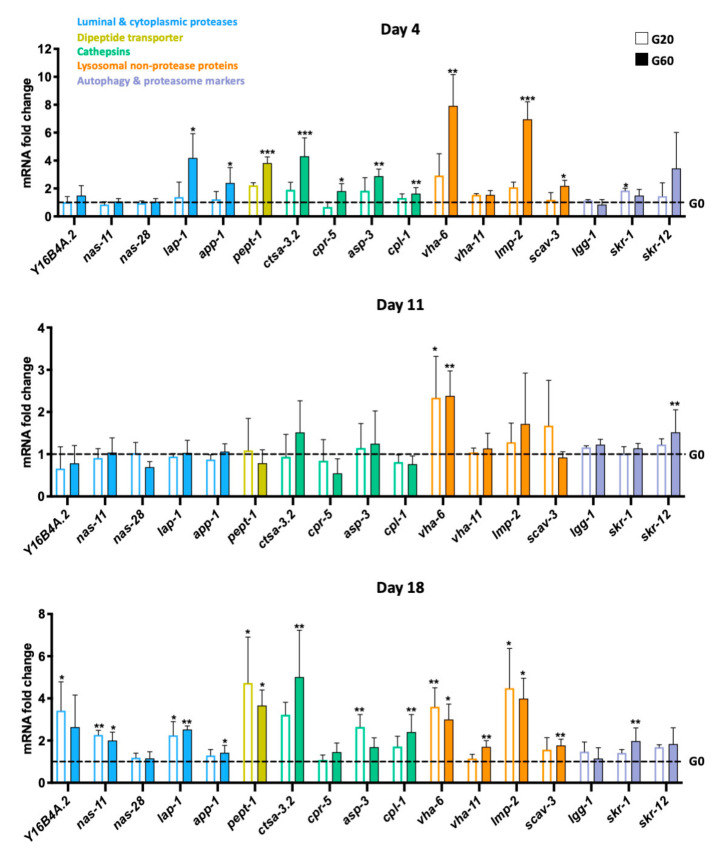
Impact of dietary *Nε*-carboxymethyllysine (dCML) exposure on transcriptional regulation of some digestive enzymes, lysosomal proteins and proteasomal and autophagy markers. Young adult worms were fed with bacteria, which were pretreated with 0 mM (G0), 20 mM (G20) or 60 mM (G60) of glyoxylic acid. After 4, 11 and 18 days of culture, relative expression of genes coding for luminal and/or cytoplasmic proteases, cathepsins, lysosomal nonprotease proteins, an autophagy marker and proteasome markers were analyzed by comparing all the conditions to the G0. Data were expressed as means ± standard deviation of 3 biological replicates. The *cdc-42* and *pmp-3* genes were used to normalize levels of gene expression. * *p* ≤ 0.05, ** *p* ≤ 0.01, *** *p* ≤ 0.001 G20 or G60 vs. G0 (Kruskal–Wallis, Dunn’s multiple comparisons test).

Concerning the intestinal digestion of glycated proteins, we checked whether we could detect different transcriptional profiles between the worms exposed or not to dCML. We have selected genes encoding different intestinal proteases: members of the astacin family of metalloproteases (nematode astacin proteases 11 and 28, NAS-11 and NAS-28), aminopeptidases (leucine aminopeptidase 1 and aminopeptidase P 1, LAP-1 and APP-1), a carboxypeptidase (Y16B4A.2) and cathepsins A (cathepsin A homolog 3.2, CTSA-3.2), B (cysteine protease related 5, CPR-5), D (aspartyl protease 3, ASP-3) and L (cathepsin L family 1, CPL-1). Among the genes encoding the luminal or cytoplasmic digestive proteases, only *lap-1* and *app-1* were significantly overexpressed on days 4 and 18 under glycating conditions, mainly with the highest concentration of dCML (Figure 3). These results show that worms do not have a major transcriptional reprogramming of their digestive enzymes when they were grown in CML-rich diets. We checked the digestion in worms incubated for 4 days with the control bacteria or the most glycated bacteria (G60) or a mixture with different ratios of these two bacteria. After 4 days of culture, the worms were incubated for 1 day in the medium without bacteria. Immunostaining of the worm lysates, compared to those fed for 4 days with the bacteria, shows a major decrease in CML epitopes, suggesting that glycated bacterial proteins were digested, at least up to the detection threshold of glycated peptides in western blot (Figure S2). Very few glycated proteins had been poorly digested.

The upregulation of the genes encoding the aminopeptidases in glycated conditions is very interesting, especially *app-1* which codes for a cytoplasmic aminopeptidase. APP-1 could be more expressed to hydrolyze the glycated di-tripeptides transported in the cytosol by PEPT-1. As PEPT-1 is transcriptionally regulated by its substrates [57], we checked expression of its gene. Our data show that *pept-1* was indeed overexpressed at the same times and under the same conditions as *app-1* (Figure 3). Most of the cathepsin encoding genes were overexpressed on days 4 and 18 in worms exposed to dCML, especially G60 (Figure 3). As cathepsins are lysosomal proteases, our results suggest that worms need lysosomes and its hydrolases to digest glycated bacteria. We therefore investigated the expression of lysosomal genes encoding non-protease proteins. Among the genes tested we chose those encoding membrane proteins, the lysosome-membrane associated protein LMP-2 (mammalian LAMP1) and the scavenger receptor 3 (SCAV-3) which maintains lysosomal integrity, as well as components of the vacuolar-type ATPase (V-ATPase) proton pump (VHA-6 and -11) which are essential to regulate the lysosomal pH to promote lysosomal hydrolase activity. Most of these genes were overexpressed in the presence of dCML, in particular G60 on days 4 and 18. These results suggest 1—an increase in the lysosomal activity of worms exposed to dCML and 2—endocytosis of glycated proteins, potentially after luminal digestion. We were surprised to find that most of the lysosomal genes were not regulated on day 11. By analyzing the kinetics of the expression of these genes in the control condition, we noticed that most of these genes were significantly, or tended to be, more expressed at this time, compared to day 4, like most of the genes analyzed coding for digestive enzymes (Figure S3). The overexpression of these genes on day 11 certainly compensated for their need in glycating conditions before their expression was again increased on day 18.

As endocytosis has a common denominator, ubiquitin, in the targeting of substrates with two other degradation pathways, the proteasome and autophagy [58], we analyzed the regulation of the expression of markers of these processes: the genes encoding two components of the ubiquitin ligase complex (proteasome), SKR-1 and SKR-12, and the gene encoding an autophagy-related protein, LGG-1 (Figure 3). Unlike the expression of *lgg-1* which was not modulated by any diet at the three incubation times, the expression of *skr-1* and *skr-12* was significantly higher, or at least tended to be, after 4 and 18 days of culture of the worms with the glycated diets. These results suggest that the activity of the proteasome would be higher after exposure to dCML, possibly following its endocytosis.

### 3.6. CML-Bound Proteins Are Endocytosed by the Worms’ Enterocytes

**Figure 4:**
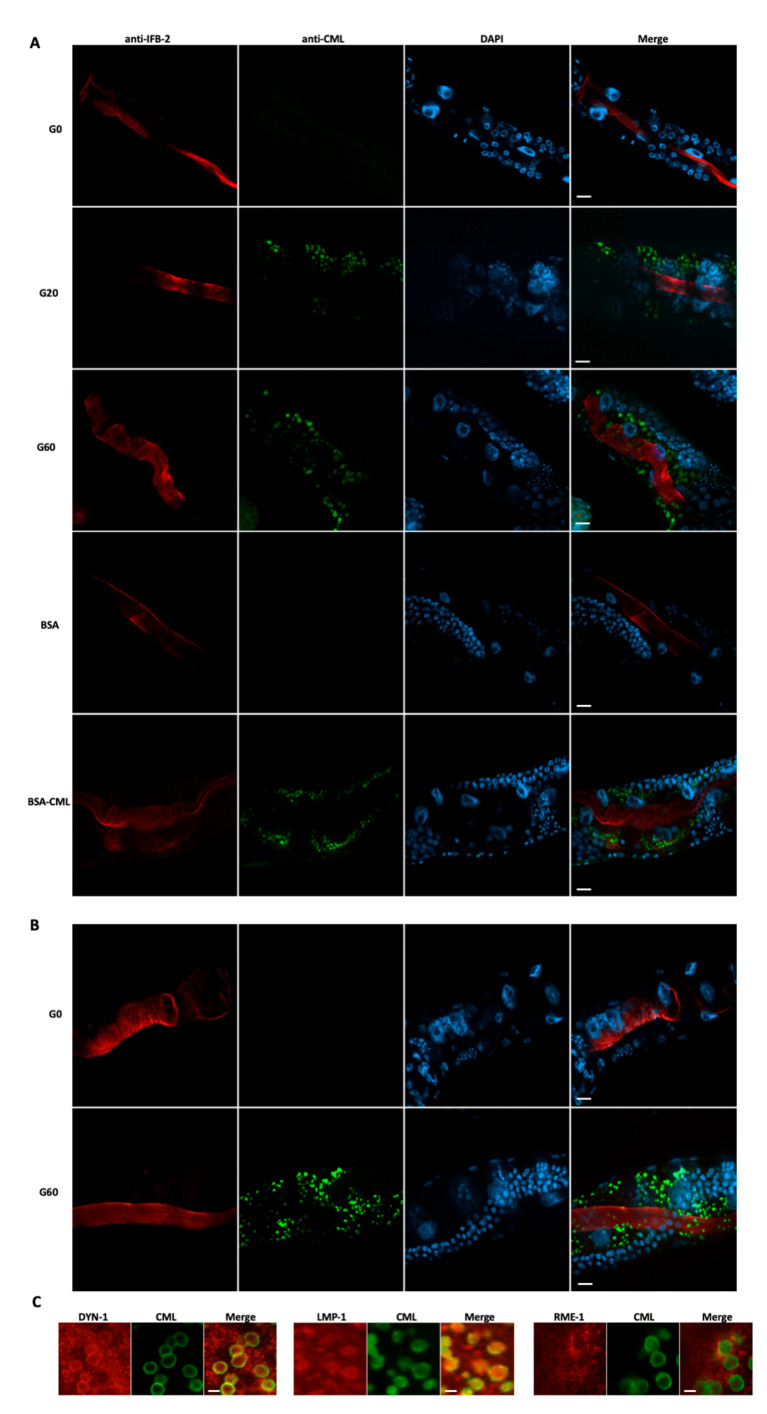
Mapping of *Nε*-carboxymethyllysine (CML) epitopes in *C. elegans* intestine. (**A**) Immunohistochemical staining was performed on worms fed for 4 days with bacteria, which were pretreated with 0 mM (G0), 20 mM (G20) or 60 mM (G60) of glyoxylic acid, or 1.6 mg/mL of non-glycated bovine serum albumin (BSA) or glycated BSA (BSA-CML), using anti-CML and anti-IFB-2 antibodies. (**B**) Worms were fed for 4 days with either G0 or G60 bacteria. After washing, worms were incubated for 6 h in nutrient-free buffer, fixed and permeabilized with modified Bouin’s solution and stained with anti-CML and anti-intermediate filament B (IFB-2) antibodies. (**C**) Immunohistochemical staining was performed on worms fed for 4 days with G60 using either anti-CML and anti-dynamin (DYN-1) antibodies, anti-CML and anti-lysosome-associated membrane protein homolog 1 (LMP-1) antibodies or anti-CML and anti-endocytosis-mediating receptor 1 (RME-1) antibodies. Worms’ nuclei were stained with DAPI. For each type of feeding condition, fluorescence micrographs with the mentioned DNA-binding probe or antibody are shown. Scale bars = 10 μm (**A**,**B**) and 2.5 μm (**C**). All results are representative of 3 independent experiments.

In order to confirm the endocytosis of CML-bound proteins or peptides, we performed immunohistochemical staining with the anti-CML antibody. In order to optimize the detection of CML epitopes inside enterocytes, we followed with slight adaptations a protocol eliminating luminal epitopes [52]. To locate the apical cortex of enterocytes, we co-stained the worms with an antibody specific for an intermediate filament protein of this cortex, IFB-2. Several vesicles, most probably endosomes, labeled with the anti-CML antibody were observed around the nuclei of the enterocytes, only of the worms incubated with both glycated bacteria, G20 and G60 (Figure 4A). More than 50 and 80% of worms contained CML-labeled endosomes after 1 and 4 days of incubation with glycated bacteria, respectively (data not shown). In order to verify if dCML endocytosis depends on the glycated protein, we analyzed whether a glycated supplement protein (BSA) could be endocytosed. We, therefore, cultured the worms with non-glycated bacteria containing a protein supplement, BSA, glycated or non-glycated. Immunohistochemistry with the anti-CML antibody showed that the worms grown with BSA-CML had endosomes containing CML epitopes (Figure 4A). As expected, the worms incubated with the BSA control were negative. These results show that any CML-bound protein could be endocytosed by *C. elegans* enterocytes.

We followed the evolution of the CML epitopes by washing the worms fed for 4 days with the most glycated bacteria (G60) and by incubating them for 6 h in M9 buffer without food. After immunohistochemical observation of the worms, some endosomes containing CML epitopes appeared smaller, suggesting endolysosomal degradation of the CML-bound peptides (Figure 4B).

To confirm endocytosis and the fusion of endosomes with lysosomes, we performed a series of double immunostaining with anti-CML antibody and antibodies recognizing different markers of endocytosis: dynamin, DYN-1, which is involved in the early phase of endocytosis; the lysosome-associated membrane protein LMP-1 (mammalian LAMP1) and an endocytosis-mediating receptor, RME-1, which is involved in the process of endosome recycling. The results show a co-localization between the CML epitopes and the DYN-1 and LMP-1 epitopes, confirming the process of endocytosis of dCML and the fusion of endosomes with lysosomes, respectively (Figure 4C). Some CML epitopes are localized at the periphery of endosomes.

### 3.7. Endocytosis of CML-Bound Bacterial Proteins Is Specific of CML

**Figure 5:**
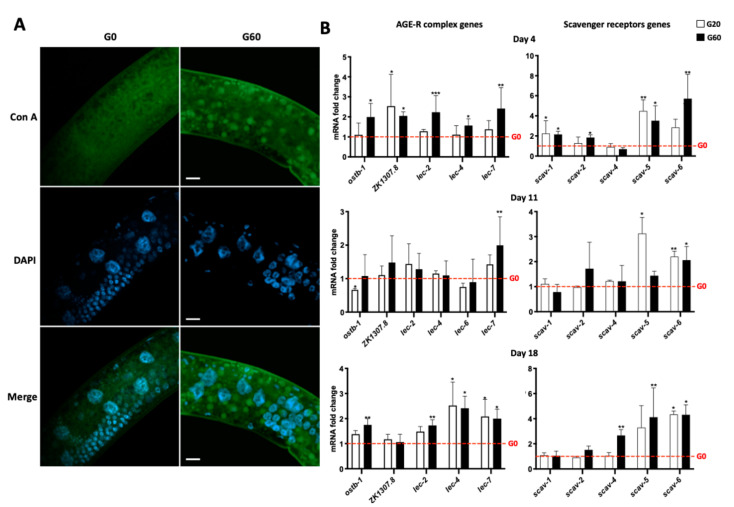
Specificity of endocytosis for dietary *Nε*-carboxymethyllysine and analysis of expression of advanced glycation end product (AGE) receptor encoding genes. (**A**) Worms fed for 4 days with bacteria, which were pretreated with 0 mM (G0) or 60 mM (G60) of glyoxylic acid, were fixed and permeabilized with modified Bouin’s solution and stained with concanavalin A (Con A). Worms’ nuclei were stained with DAPI. For each type of feeding condition, fluorescence micrograph with the mentioned DNA-binding probe or lectin are shown. Scale bars = 10 μm. Endogenous glycoproteins were stained with Con A, a diffuse labeling is visible in the worms incubated with the control bacteria. All results are representative of 3 independent experiments. (**B**) Young adult worms were fed with G0, G20 (bacteria pretreated with 20 mM of glyoxylic acid) and G60. After 4, 11 and 18 days of culture, relative expression of mammalian ortholog coding for members of AGE-R complex and class A and B scavenger receptors, was analyzed by comparing all the conditions to the G0. Data were expressed as means ± standard deviation of 3 biological replicates. The *cdc-42* and *pmp-3* genes were used to normalize levels of gene expression. * *p* ≤ 0.05, ** *p* ≤ 0.01, *** *p* ≤ 0.001 G20 or G60 vs. G0 for the same incubation time (Kruskal–Wallis, Dunn’s multiple comparisons test).

We wondered if the endocytosis was specific for glycated proteins. We, therefore, labeled the worms with Con A which recognized both non-glycated and glycated bacteria (Figure 1A). *C. elegans* has glycoproteins that can be labeled with Con A, but none has been identified as marker of endolysosomes. Moreover, fluorescent Con A did not stain the endosomes of worms incubated with the non-glycated bacteria, suggesting that the non-glycated bacteria molecules were not endocytosed (Figure 5A). In contrast, worms incubated with glycated bacteria contained endosomes labeled with Con A. These endosomes co-stained with LMP-1 epitopes (Figure S4). The endocytosis of glycated bacterial components therefore seems to depend on CML. There could be co-endocytosis of non-glycated bacterial compounds with CML-bound bacterial proteins or peptides. The hypothesis of endocytosis of CML-bound peptides/proteins mediated by an AGE receptor seemed to be quite plausible. As *C. elegans* has different orthologs of the human genes encoding AGE receptors except RAGE, we analyzed the expression of these orthologs in the presence of glycated or non-glycated bacteria. We measured the relative expression of the orthologs of human genes encoding 1—the AGE-R complex: OST-B1 (mammalian AGE-R1), ZK1307.8 (mammalian AGE-R2) and the members of the galectin (LEC) family (mammalian AGE-R3/galectin-3) and 2—the members of the SCAV family (mammalian SR-A and SR-B members). On day 4, the two glycated diets induced significantly the expression of three of these genes: the genes coding for SCAV-1, SCAV-5 and ZK1307.8 (Figure 5B). Although not significantly, *scav-6* expression was increased by the two glycated regimens. From these genes, only the expression of *scav-5* and *scav-6* was significantly increased by at least one glycated diet on days 11 and 18. On day 18, the genes encoding LEC-4 and LEC-7 were more expressed in the presence of dCML, whatever its concentration. These data suggest a possible role of some of these receptors, notably SCAV-5, in dCML mediated endocytosis.

## 4. Discussion

Clearance of AGEs occurs in the endothelium, vasculature, neural tissues and some types of immune cells, mainly by endocytosis [3,16,59]. Various AGE receptors, including SR-A and SR-B members and the AGE-R complex, induce this process. With regard to dAGEs, this mechanism would take place after intestinal absorption and passage into the blood vessels. It is surprising that endocytosis of AGEs by intestinal cells, especially enterocytes, has not been further considered. On the one hand, SR-A and SR-B members and AGE-R complex are indeed expressed by enterocytes (https://www.proteinatlas.org, accessed on 6 December 2021), in particular in the small intestine, and on the other hand, endocytosis in the enterocytes is an essential cellular process with various biological impacts, including digestion and absorption of nutrients [60]. Until now, only the passage of free AGE adducts or glycated di-tripeptides into enterocytes has been reported. Uptake of these AGEs involves an oligopeptide transporter, PEPT-1, or passive diffusion [39,40]. The glycated di-tripeptides are further digested by endopeptidases in the enterocytes before the free AGE adducts are released into the circulation. In our study, ingestion of glycated bacteria induced the expression of *pept-1*, *C. elegans* ortholog to the human SLC15A1 gene coding for PEPT-1. Since PEPT-1 is transcriptionally regulated by its substrates [57], we suspect that a similar regulatory process is taking place in *C. elegans*. Indeed, the ingestion of CML-bound proteins by the worms has probably generated an accumulation of glycated di-tripeptides, requiring transport by PEPT-1 for their absorption by enterocytes. After exposure of the worms to dCML, we also observed the induction of a gene encoding a cytoplasmic endopeptidase, APP-1, potentially involved in the digestion of glycated di-tripeptides. These results support our decision to use *C. elegans* as a relevant model to analyze the digestion and absorption of glycated peptides.

While simple diffusion and transport by PEPT-1 are undoubtedly two absorption pathways for dAGEs, others must take place to further explain certain pathophysiological mechanisms described or hypothesized for dAGEs [2,3,12]. For example, the mechanisms involving RAGE can be activated only by glycated proteins or larger peptides than di-tripeptides [36]. One of the most plausible hypotheses to date would be that dAGEs induce the formation of endogenous AGEs which would bind to RAGE to activate different signaling pathways. Some studies have actually shown that endogenous AGEs are produced following the ingestion of dAGEs including dCML [29]. Our results show that, indeed, dCML significantly induced the expression of genes encoding glyoxalases which convert endogenous glycation precursors, α-ketoaldehydes such as glyoxal and methylglyoxal, into α-hydroxyacids [56]. Oxidizing conditions favor the generation of these precursors. The expression of *sod-3* and *gst-4* genes, key markers of the antioxidant response in *C. elegans*, were indeed significantly higher throughout the experiment when the worms were incubated with glycated bacteria, especially the most glycated one. Induction of these markers by dCML shows that the latter is certainly a source of reactive oxygen species which would induce the expression of these genes and potentially the production of α-ketoaldehydes. The increase in proteasomal markers (*skr-1* and *skr-12*) in worms exposed to dCML could be a cellular response to degrade the endogenous AGEs formed. Additional experiments, in particular the analysis of endogenous AGEs and other proteasomal markers, would allow this hypothesis to be verified.

Bacteria are the main food for worms. Their glycation, even if it did not impair membrane and the parietal integrity of the bacteria, could have induced a nutritional alteration of this food with potential physiological effects. We have, indeed, detected a reproduction defect in worms incubated with glycated bacteria. These worms had fewer embryos and laid fewer eggs than worms under the normal diet. Additional analyses will of course be necessary to determine the pathophysiological mechanisms involved following ingestion of dCML. We suspected that the glycated diets could have induced a state of starvation leading to the reproduction defect. However, starvation has been shown to have only a small impact on the number of eggs laid by the worms [61]. In addition, our results show that the expression of *trx-1*, which is induced by starvation [54], was not modulated by glycated diets at least up to 11 days of culture. Likewise, the gene encoding a key autophagy molecule, LGG-1, has never been regulated by dCML. Autophagy is indeed an essential mechanism allowing, among other things, energetic homeostasis to be maintained during starvation [62]. An absence of starvation does not, however, exclude a possible nutritional impact of food glycation, which should be explored.

The exposure of the worms to dCML clearly caused cellular alterations as evidenced on the one hand by the induction of the antioxidant response but also the induction of the cytoplasmic and mitochondrial unfolded protein responses (UPR^cyt^ and UPR^mt^, respectively), in particular the significant increase in the genes encoding the chaperones HSP-70 and HSP-6, respectively. Any causal or consequential link between the reproductive defect in worms exposed to dCML and the induction of antioxidant and unfolded protein responses remains to be determined. Among the genes encoding digestive enzymes, *lap-1*, which codes for an endopeptidase, was induced by the two glycated diets on days 4 and 18. Inhibition of *lap-1* was described to lead to a defect in oviposition [63]. The increased expression of *lap-1* could then be a response to the reproductive defect induced by *lap-1*.

We were surprised to discover, among the genes whose expression was induced by dCML, the genes encoding lysosomal proteases, cathepsins. The expression of other lysosomal genes was increased in CML-rich diets, such as those encoding V-ATPase, which regulate the pH of lysosomes to promote cathepsin activity, and the lysosome-associated membrane protein homolog 2 (LMP-2 or LAMP1 in mammals), which is important for the functionality of lysosomes. These results strongly suggest that dCML has been internalized by enterocytes and has undergone intracellular hydrolysis. The glycated di-tripeptides transported by PEPT-1 are effectively hydrolyzed before the free AGE adducts pass into the general circulation. However, this digestion involves cytoplasmic but not lysosomal endopeptidases [39]. The hypothesis of endocytosis of glycated proteins or larger peptides was therefore verified by immunohistochemistry. The images clearly show the presence of endosomes containing CML epitopes in the worms. These endosomes presented distinct markers such as dynamin 1 or DYN-1, which is involved in the early stage of endocytosis, the lysosomal membrane protein LMP-1 which confirms fusion with lysosomes and, for some, the RME-1 receptor which is involved in endosomal recycling. These results clearly demonstrate a new mechanism of intestinal absorption for dCML which is therefore endocytosis.

As mentioned earlier, what is most surprising is not to have demonstrated endocytosis as a route of intestinal absorption of dAGEs but rather not to have demonstrated it earlier. The absorption of AGEs has mainly been analyzed in vitro with cells of an immortalized cell line of human colorectal adenocarcinoma cells named Caco-2, which are commonly used to study intestinal permeability [39,40,64]. Because these epithelial cells are derived from colon cells, they do not have all of the characteristics of small intestine enterocytes, including the expression of digestive enzymes in their microvilli [65]. In addition, absorption experiments were mostly performed with small defined glycated oligopeptides instead of glycated proteins or larger peptides. In *C. elegans*, it was possible to carry out immunohistochemical analysis while preserving the whole intestine of the worm. In addition, the worm’s intestine contains only enterocytes, making visualization of these cells easy. In mammalian models, such as the murine ones, the intestinal epithelium can only be analyzed by cell imaging after histological sections. Since it is composed of several cell types, without electron microscopy, the analysis is much more complex than in the worm. This once again demonstrates the essential contribution of so-called simple model organisms to analyze certain cellular functions.

The use of *C. elegans* to analyze the absorption of dAGEs by enterocytes is all the more relevant as this organism expresses the orthologs of the genes encoding the AGE receptors described for endocytozing AGEs in non-intestinal tissues: SR-A and SR-B members and the AGE-R complex. Analysis of the expression of these genes after culturing the worms with non-glycated or glycated bacteria showed a stronger expression of the genes encoding SCAV-5, SCAV-6 and some LECs in the presence of glycated bacteria, regardless of their glycation rate. These receptors may mediate the endocytosis of dCML. Our immunohistochemical staining with Con A revealed that the endocytosis of bacterial components was indeed specific for dCML. By targeting these genes by RNA interference in a future study, we will be able to explore the role of these receptors in the endocytosis of dCML.

Endocytosis is a mechanism that can modulate different cellular functions and participate in cellular adaptation to different stresses, but it is an active process that consumes energy in the form of adenosine triphosphate (ATP) [66,67]. The enterocytes of worms incubated with glycated bacteria, therefore, certainly need more ATP than those of worms incubated with non-glycated bacteria. They must therefore produce more ATP from various catabolic mechanisms including respiration in the mitochondrial matrix. This need can lead to ATP deficiencies for other important cellular functions, including certain enzymatic activities. In addition, increased mitochondrial respiration can be a source of oxidative stress. This could explain on the one hand the increase in antioxidant responses including the expression of the mitochondrial SOD-3 encoding gene and that of the gene encoding the HSP-6 chaperone of UPR^mt^ with impacts on cytoplasmic homeostasis. Since bacteria are the main food source for worms, the nutritional intake of glycated bacteria might not compensate for the energy required by the endocytosis of glycated proteins. The reproductive defect of worms incubated with dCML might be therefore associated with the ATP shortage induced by endocytosis.

Our study deserves additional analyses, in particular: 1—the quantification of the fate of dCML (free CML adduct and CML-bound protein) ingested by worms, 2—deciphering between endocytosed dCML and dCML transported by PEPT-1, 3—a demonstration of the health effect of glycated dietary proteins vs. glycated protein supplement which, as we have shown in this study, is also endocytosed by enterocytes, 4—highlighting the mechanisms induced by the ingestion of dCML and the receptors involved. It is essential to analyze the fate of endocytosed dCML, in particular its digestion and/or its possible transcytosis in the lamina propria. Exocytosis of glycated peptides could promote food intolerance and activate RAGE in other organs after transport via the general circulation. *C. elegans* is a good model for studying the health effects of dAGEs; not necessarily for the observed phenotypes which are quite dependent on the organism studied but for the induced mechanisms which are often conserved in the animal kingdom.

## 5. Conclusions

Our results have given new insights into the intestinal absorption of dCML. We have indeed shown that dCML was endocytosed by enterocytes. In parallel, we have observed a reproductive defect in worms exposed to dCML. Additional studies will be necessary to determine the mechanisms, possibly dependent on dCML endocytosis, involved in this pathophysiological effect. However, our results are already opening up new perspectives to better characterize the pathophysiological mechanisms of dAGEs.

## Supplementary Materials

The following are available online at https://www.mdpi.com/article/10.3390/nu13124398/s1, Table S1: Primers’ list, Figure S1: Relative expression of *hsp-16.2* gene, Figure S2: Digestion of bacterial glycated proteins by *C. elegans*, Figure S3: Impact of dietary *Nε*-carboxymethyllysine (dCML) exposure on transcriptional regulation of some digestive enzymes and lysosomal proteins, Figure S4: Mapping of lysosomal epitope and concanavalin A (Con A) staining on intestinal endosomes.

## References

[1] Sharma C., Kaur A., Thind S.S., Singh B., Raina S. (2015). Advanced glycation End-products (AGEs): An emerging concern for processed food industries. J. Food Sci. Technol..

[2] Nowotny K., Schröter D., Schreiner M., Grune T. (2018). Dietary advanced glycation end products and their relevance for human health. Ageing Res. Rev..

[3] Zhang Q., Wang Y., Fu L. (2020). Dietary advanced glycation end-products: Perspectives linking food processing with health implications. Compr. Rev. Food Sci. Food Saf..

[4] Di Pino A., Currenti W., Urbano F., Mantegna C., Purrazzo G., Piro S., Purrello F., Rabuazzo A.M. (2016). Low advanced glycation end product diet improves the lipid and inflammatory profiles of prediabetic subjects. J. Clin. Lipidol..

[5] Lopez-Moreno J., Quintana-Navarro G.M., Delgado-Lista J., Garcia-Rios A., Alcala-Diaz J.F., Gomez-Delgado F., Camargo A., Perez-Martinez P., Tinahones F.J., Striker G.E. (2016). Mediterranean Diet Supplemented With Coenzyme Q10Modulates the Postprandial Metabolism of Advanced Glycation End Products in Elderly Men and Women. J. Gerontol. Ser. A Boil. Sci. Med. Sci..

[6] Macías-Cervantes M.H., Rodríguez-Soto J.M.D., Uribarri J., Díaz-Cisneros F.J., Cai W., Garay-Sevilla M.E. (2015). Effect of an advanced glycation end product-restricted diet and exercise on metabolic parameters in adult overweight men. Nutrition.

[7] Tantalaki E., Piperi C., Livadas S., Kollias A., Adamopoulos C., Koulouri A., Christakou C., Diamanti-Kandarakis E. (2014). Impact of dietary modification of advanced glycation end products (AGEs) on the hormonal and metabolic profile of women with polycystic ovary syndrome (PCOS). Hormones.

[8] Uribarri J., Cai W., Ramdas M., Goodman S., Pyzik R., Chen X., Zhu L., Striker G.E., Vlassara H. (2011). Restriction of Advanced Glycation End Products Improves Insulin Resistance in Human Type 2 Diabetes. Diabetes Care.

[9] Harcourt B.E., Sourris K.C., Coughlan M.T., Walker K.Z., Dougherty S.L., Andrikopoulos S., Morley A.L., Thallas-Bonke V., Chand V., Penfold S.A. (2011). Targeted reduction of advanced glycation improves renal function in obesity. Kidney Int..

[10] Luévano-Contreras C., Garay-Sevilla M.E., Wrobel K., Malacara J.M., Wrobel K. (2013). Dietary advanced glycation end products restriction diminishes inflammation markers and oxidative stress in patients with type 2 diabetes mellitus. J. Clin. Biochem. Nutr..

[11] Yacoub R., Nugent M., Cai W., Nadkarni G.N., Chaves L.D., Abyad S., Honan A.M., Thomas S.A., Zheng W., Valiyaparambil S.A. (2017). Advanced glycation end products dietary restriction effects on bacterial gut microbiota in peritoneal dialysis patients; a randomized open label controlled trial. PLoS ONE.

[12] Poulsen M.W., Hedegaard R.V., Andersen J.M., de Courten B., Bügel S., Nielsen J., Skibsted L.H., Dragsted L.O. (2013). Advanced glycation endproducts in food and their effects on health. Food Chem. Toxicol..

[13] Birlouez-Aragon I., Saavedra G., Tessier F., Galinier A., Ait-Ameur L., Lacoste F., Niamba C.-N., Alt N., Somoza V., Lecerf J.-M. (2010). A diet based on high-heat-treated foods promotes risk factors for diabetes mellitus and cardiovascular diseases. Am. J. Clin. Nutr..

[14] Semba R.D., Gebauer S.K., Baer D.J., Sun K., Turner R., Silber H.A., Talegawkar S., Ferrucci L., Novotny J.A. (2014). Dietary Intake of Advanced Glycation End Products Did Not Affect Endothelial Function and Inflammation in Healthy Adults in a Randomized Controlled Trial. J. Nutr..

[15] Semba R.D., Ang A., Talegawkar S., Crasto C., Dalal M., Jardack P., Traber M., Ferrucci L., Arab L. (2011). Dietary intake associated with serum versus urinary carboxymethyl-lysine, a major advanced glycation end product, in adults: The Energetics Study. Eur. J. Clin. Nutr..

[16] Poulsen M.W., Bak M.J., Andersen J.M., Monošík R., Giraudi-Futin A.C., Holst J.J., Nielsen J., Lauritzen L., Larsen L.H., Bügel S. (2014). Effect of dietary advanced glycation end products on postprandial appetite, inflammation, and endothelial activation in healthy overweight individuals. Eur. J. Nutr..

[17] Reddy M.B., Love M. (1999). The Impact of Food Processing on the Nutritional Quality of Vitamins and Minerals. Adv. Exp. Med. Biol..

[18] Tessier F.J., Birlouez-Aragon I. (2010). Health effects of dietary Maillard reaction products: The results of ICARE and other studies. Amino Acids.

[19] Scheijen J.L., Clevers E., Engelen L., Dagnelie P.C., Brouns F., Stehouwer C.D., Schalkwijk C.G. (2016). Analysis of advanced glycation endproducts in selected food items by ultra-performance liquid chromatography tandem mass spectrometry: Presentation of a dietary AGE database. Food Chem..

[20] Feng J., Hou F., Liang M., Wang G., Zhang X., Li H., Xie D., Tian J., Liu Z. (2007). Restricted intake of dietary advanced glycation end products retards renal progression in the remnant kidney model. Kidney Int..

[21] Hofmann S.M., Dong H.-J., Li Z., Cai W., Altomonte J., Thung S.N., Zeng F., Fisher E.A., Vlassara H. (2002). Improved Insulin Sensitivity Is Associated With Restricted Intake of Dietary Glycoxidation Products in the db/db Mouse. Diabetes.

[22] Guan S.-S., Sheu M.-L., Yang R.-S., Chan D.-C., Wu C.-T., Yang T.-H., Chiang C.-K., Liu S.-H. (2016). The pathological role of advanced glycation end products-downregulated heat shock protein 60 in islet β-cell hypertrophy and dysfunction. Oncotarget.

[23] Lima M.T.N.S., Howsam M., Anton P.M., Delayre-Orthez C., Tessier F.J. (2021). Effect of Advanced Glycation End-Products and Excessive Calorie Intake on Diet-Induced Chronic Low-Grade Inflammation Biomarkers in Murine Models. Nutrients.

[24] Patel R., Baker S.S., Liu W., Desai S., Alkhouri R., Kozielski R., Mastrandrea L., Sarfraz A., Cai W., Vlassara H. (2012). Effect of Dietary Advanced Glycation End Products on Mouse Liver. PLoS ONE.

[25] Lubitz I., Ricny J., Atrakchi-Baranes D., Shemesh C., Kravitz E., Liraz-Zaltsman S., Maksin-Matveev A., Cooper I., Leibowitz A., Uribarri J. (2016). High dietary advanced glycation end products are associated with poorer spatial learning and accelerated Aβ deposition in an Alzheimer mouse model. Aging Cell.

[26] Cai W., Uribarri J., Zhu L., Chen X., Swamy S., Zhao Z., Grosjean F., Simonaro C., Kuchel G., Schnaider-Beeri M. (2014). Oral glycotoxins are a modifiable cause of dementia and the metabolic syndrome in mice and humans. Proc. Natl. Acad. Sci. USA.

[27] Lund M.N., Ray C. (2017). Control of Maillard Reactions in Foods: Strategies and Chemical Mechanisms. J. Agric. Food Chem..

[28] Oh N.S., Joung J.Y., Lee J.Y., Song J.G., Oh S., Kim Y., Kim H.W., Kim S.H. (2020). Glycated milk protein fermented with Lactobacillus rhamnosus ameliorates the cognitive health of mice under mild-stress condition. Gut Microbes.

[29] Grossin N., Auger F., Niquet-Leridon C., Durieux N., Montaigne D., Schmidt A.M., Susen S., Jacolot P., Beuscart J.-B., Tessier F. (2015). Dietary CML-enriched protein induces functional arterial aging in a RAGE-dependent manner in mice. Mol. Nutr. Food Res..

[30] Rupa P., Mine Y. (2019). Comparison of Glycated Ovalbumin–Monosaccharides in the Attenuation of Ovalbumin-Induced Allergic Response in a BALB/C Mouse Model. J. Agric. Food Chem..

[31] Chuyen N., Arai H., Nakanishi T., Utsunomiya N. (2005). Are Food Advanced Glycation End Products Toxic in Biological Systems?. Ann. N. Y. Acad. Sci..

[32] Miller A., Jedrzejczak W.W. (2001). Albumin-biological functions and clinical significance. Postepy Hig. Med. Dosw..

[33] Shimizu M. (2004). Food-derived peptides and intestinal functions. BioFactors.

[34] Bettiga A., Fiorio F., Di Marco F., Trevisani F., Romani A., Porrini E., Salonia A., Montorsi F., Vago R. (2019). The Modern Western Diet Rich in Advanced Glycation End-Products (AGEs): An Overview of Its Impact on Obesity and Early Progression of Renal Pathology. Nutrients.

[35] Hellwig M., Matthes R., Peto A., Löbner J., Henle T. (2014). N-ε-fructosyllysine and N-ε-carboxymethyllysine, but not lysinoalanine, are available for absorption after simulated gastrointestinal digestion. Amino Acids.

[36] Xue J., Rai V., Singer D., Chabierski S., Xie J., Reverdatto S., Burz D.S., Schmidt A.M., Hoffmann R., Shekhtman A. (2011). Advanced Glycation End Product Recognition by the Receptor for AGEs. Structure.

[37] Tuma P.L., Hubbard A.L. (2003). Transcytosis: Crossing Cellular Barriers. Physiol. Rev..

[38] Vaziri N.D., Yuan J., Nazertehrani S., Ni Z., Liu S. (2013). Chronic Kidney Disease Causes Disruption of Gastric and Small Intestinal Epithelial Tight Junction. Am. J. Nephrol..

[39] Hellwig M., Geissler S., Matthes R., Peto A., Silow C., Brandsch M., Henle T. (2011). Transport of Free and Peptide-Bound Glycated Amino Acids: Synthesis, Transepithelial Flux at Caco-2 Cell Monolayers, and Interaction with Apical Membrane Transport Proteins. ChemBioChem.

[40] Grunwald S., Krause R., Bruch M., Henle T., Brandsch M. (2006). Transepithelial flux of early and advanced glycation compounds across Caco-2 cell monolayers and their interaction with intestinal amino acid and peptide transport systems. Br. J. Nutr..

[41] Liang Z., Chen X., Li L., Li B., Yang Z. (2020). The fate of dietary advanced glycation end products in the body: From oral intake to excretion. Crit. Rev. Food Sci. Nutr..

[42] Smith P.K., Masilamani M., Li X.-M., Sampson H.A. (2017). The false alarm hypothesis: Food allergy is associated with high dietary advanced glycation end-products and proglycating dietary sugars that mimic alarmins. J. Allergy Clin. Immunol..

[43] Gupta R.K., Gupta K., Sharma A., Das M., Ansari I.A., Dwivedi P.D. (2018). Maillard reaction in food allergy: Pros and cons. Crit. Rev. Food Sci. Nutr..

[44] Ullah M.A., Loh Z., Gan W.J., Zhang V., Yang H., Li J.H., Yamamoto Y., Schmidt A.M., Armour C.L., Hughes J.M. (2014). Receptor for advanced glycation end products and its ligand high-mobility group box-1 mediate allergic airway sensitization and airway inflammation. J. Allergy Clin. Immunol..

[45] Xu S., Li L., Zhengyang B., Ye F., Shao C., Sun Z., Bao Z., Dai Z., Zhu J., Jing L. (2018). CML/CD36 accelerates atherosclerotic progression via inhibiting foam cell migration. Biomed. Pharmacother..

[46] Zhuang A., Yap F.Y., Bruce C., Leung C., Plan M.R., Sullivan M., Herath C., McCarthy D., Sourris K.C., Kantharidis P. (2017). Increased liver AGEs induce hepatic injury mediated through an OST48 pathway. Sci. Rep..

[47] Snelson M., Tan S.M., Thallas-Bonke V., Sourris K., Ziemann M., El-Osta A., Cooper M., Forbes J., Coughlan M. (2021). Thermally Processed Diet-Induced Albuminuria, Complement Activation and Intestinal Permeability Are Attenuated by Resistant Starch in Experimental Diabetes. Curr. Dev. Nutr..

[48] Alsamad F., Brunel B., Vuiblet V., Gillery P., Jaisson S., Piot O. (2021). In depth investigation of collagen non-enzymatic glycation by Raman spectroscopy. Spectrochim. Acta Part A Mol. Biomol. Spectrosc..

[49] Courjol F., Jouault T., Mille C., Hall R., Maes E., Sendid B., Mallet J.M., Guerardel Y., Gow N.A.R., Poulain D. (2015). β-1,2-Mannosyltransferases 1 and 3 Participate in Yeast and Hyphae O- and N-Linked Mannosylation and Alter Candida al-bicans Fitness During Infection. Open Forum Infectious Diseases.

[50] Laemmli U.K. (1970). Cleavage of Structural Proteins during the Assembly of the Head of Bacteriophage T4. Nature.

[51] Moretton C., Gouttefangeas C., Dubois C., Tessier F.J., Fradin C., Prost-Camus E., Prost M., Haumont M., Nigay H. (2021). Investigation of the antioxidant capacity of caramels: Combination of laboratory assays and C. elegans model. J. Funct. Foods.

[52] Duerr J.S. (2006). Immunohistochemistry. WormBook.

[53] Estey T., Kang J., Schwendeman S.P., Carpenter J.F. (2006). BSA Degradation Under Acidic Conditions: A Model For Protein Instability During Release From PLGA Delivery Systems. J. Pharm. Sci..

[54] Fierro-González J.C., González-Barrios M., Miranda-Vizuete A., Swoboda P. (2011). The thioredoxin TRX-1 regulates adult lifespan extension induced by dietary restriction in *Caenorhabditis elegans*. Biochem. Biophys. Res. Commun..

[55] Thornalley P.J. (2007). Endogenous α-Oxoaldehydes and Formation of Protein and Nucleotide Advanced Glycation Endproducts in Tissue Damage. Acetaldehyde-Related Pathology: Bridging the Trans-Disciplinary Divide.

[56] He Y., Zhou C., Huang M., Tang C., Liu X., Yue Y., Diao Q., Zheng Z., Liu D. (2020). Glyoxalase system: A systematic review of its biological activity, related-diseases, screening methods and small molecule regulators. Biomed. Pharmacother..

[57] Spanier B. (2013). Transcriptional and functional regulation of the intestinal peptide transporter PEPT. J. Physiol..

[58] Clague M.J., Urbe S. (2010). Ubiquitin: Same Molecule, Different Degradation Pathways. Cell.

[59] Ott C., Jacobs K., Haucke E., Santos A.N., Grune T., Simm A. (2014). Role of advanced glycation end products in cellular signaling. Redox Biol..

[60] Zimmer K.-P., De Laffolie J., Barone M.V., Naim H.Y., Zimmer U.-P.D.K.-P. (2016). Endocytosis in enterocytes. Wien. Med. Wochenschr..

[61] Lai S., Pangilinan K., Sanjabi K., Savic D. Starvation of Adult Caenorhabditis Elegans and Its Effect on Health and Reproduc-tion. File:///C:/Users/MDPI/Downloads/184795-Article%20Text-191480-1-10-20140604.pdf.

[62] Kang C., You Y.-J., Avery L. (2007). Dual roles of autophagy in the survival of Caenorhabditis elegans during starvation. Genes Dev..

[63] Dubois C., Pophillat M., Audebert S., Fourquet P., Lecomte C., Dubourg N., Galas S., Camoin L., Frelon S. (2019). Differential modification of the C. elegans proteome in response to acute and chronic gamma radiation: Link with reproduction decline. Sci. Total Environ..

[64] Van Der Lugt T., Opperhuizen A., Bast A., Vrolijk M.F. (2020). Dietary Advanced Glycation Endproducts and the Gastrointestinal Tract. Nutrients.

[65] Lundquist P., Artursson P. (2016). Oral absorption of peptides and nanoparticles across the human intestine: Opportunities, limitations and studies in human tissues. Adv. Drug Deliv. Rev..

[66] López-Hernández T., Haucke V., Maritzen T. (2020). Endocytosis in the adaptation to cellular stress. Cell Stress.

[67] Kirchhausen T. (2012). Bending membranes. Nat. Cell Biol..

