# Supplementary material for: Exposure of Caenorhabditis elegans to Dietary Nε-Carboxymethyllysine Emphasizes Endocytosis as a New Route for Intestinal Absorption of Advanced Glycation End Products
Source: Nutrients. 2021 Dec 8;13(12):4398. doi: 10.3390/nu13124398 (PMC8705817; doi:10.3390/nu13124398)
Supplement: Supplementary file 1 [file nutrients-13-04398-s001.zip › nutrients-1463824-supplementary.pdf]

Table S1. Primers list

	Forward (5'-3')	Reverse (3'-5')
app-1	CACGAAGGACCGATCGGAAT	TAAGCACTTGGCTGGCATGA
asp-3	TGCACAACAGATTTGATTGC	AGACAACATCGTTGTCAACG
cdc-42	GATCAAGTGCGTCGTCGTTG	TGTGACGGCGTAATTGTCGA
cpl-1	GACGTCTCTTCGGTGACTCC	TGGGTATCACGCCAGTCAAC
cpr-5	TGTGTCGACTCCTGCACTTC	TGGATTTGCTCGACCTTCTT
ctsa-3.2	TTGAAACACGGGCCATGGTA	CAAGTGGCACAAAGTGACCG
djr-1.1	GCACTCTTGAGCCATGGAGT	TGCCACTGACAACAACACGA
djr-1.2	AGAGCGTGTCACTTGTCACT	ACTCTGTCGCTGATAACAACG
glod-4	GTGCCAAGACCATCGACTTT	TCTTGCTCCATCGTCCGTTAT
gst-4	TTTGATGCTCGTGCTCTTGC	AATGGGAAGCTGGCCAAATG
hsp-16.2	TGCAGAATCTCTCCATCTGAG T	TGGTTTAAACTGTGAGACGTTG A
hsp-4	AACCTACTCGTGCGTTGGAG	TCTCCGGAAAACGCAACGTA
hsp-6	TCGTGAACGTTTCAGCCAGA	CTCAGCGGCATTCTTTTCGG
hsp-70	CTACATGCAAAGCGATTGGA	GGCGTAGTCTTGTTCCCTTC
lap-1	ATTGCTCGGACCTTCTGCAA	TCCTCGTTGGTGAGCTTGTC
lec-1	TCGTTCCACTTCAACCCACG	GAATGGGTTCTTTCCCTCGC
lec-2	TGCTGAAATGGCCCATGAGT	GGTGTGAAGGTTGACGGTGA
lec-3	CCTGGAAGTGTCGTCCGTAA	TGGGTTCTTTCCCTCTCGTT
lec-4	CTCGACGCTGGACAGACATT	CACCACCTCCCTGGAGAAGA
lec-5	ATATGGCAGTTGCTGGTCTT	TTGACACGCTTTCCTTTTGG
lec-6	CGCCAATCCATTCCAGCAGA	ATGAGATGGACTCCGTGGGA
lec-7	CTCAGCTCCGACATAGCCAC	ACCGGGAACCGATGATCAAA
lec-8	GCTCAAGCACCACGATCACT	TCAACTGGGAAACGGTGTGG
lec-9	CAGGAACCTTTGCTCTCCCC	ACCGGAGATCAGGTTGACGA
lec-10	TTCACCACTCGGAGCACTTC	AATCCGATGGCTTGAACCGA
lec-11	CGTTGCAATGACTGTTCGAG	ATCGGAGTTGGAGCAACAGG
lec-12	AAAAACCGGATGGTTCAGCG	CGAATCGTTCTTCGTTGCCC
lgg-1	TACCAGGACCATCACGAGGA	CGACCTCTCCTCCATACACAC
lmp-2	TCGCTGGACTCATTGTGCTC	ATGAGCTTCTGGGTTGACCA
nas-11	GACCCTGCCAACCCAGTTTA	CGATCATTGCGAAGGTGCTG
nas-28	CAGCTGTCTGAGCTTCAAGG	AACGTATCACAGTTTGGCCC
ostb-1	AGGAGAAACTGCTGCTGTGA	ACGGTTAGCTCATGTCCTCG
pept-1	TGTCATCACGGCAGCAGAAA	AATCTCCAGCAGCAGTGGTC
pmp-3	AGTTCAGTTGCTCTCGTTGC	AGGCCAGCAAACATTCGAAG
scav-1	GTACTGCAAGGACCCGAACT	CCGACACGAATGCTGGTAGA
scav-2	AACCGCCTTTCTTGAAGTCG	TCCAAAATCGCTTCGCACAC
scav-3	TTACGATCCTGCTCTTCCGC	TGGCATCGTGACAAGTCGAT
scav-4	ACGGGTTCAAGGTGGAAGAC	ACATGGCCAGTTGGGGAAAT

scav-5	CTCAATAGCCGAGGGAGCAG	AGGATTTTTGCCGTGGGACT
scav-6	ATGCTTTACTGTCCGCTGCT	TTCGGCATAGCTGGAATCGG
skr-1	GCTGGGACGTCGAGTTTCTT	AGCAACAGTCTTGCAGGTAA
skr-12	TCCAATTGTCGATGCCCCAG	TCGATGGTGTAGCCGAGATT
sod-3	AAGCATCATGCCACCTACGT	CGCTGGTTGGAGAGCAATTG
trx-1	GAGACGCAATTGAGGCACTG	GAGCAGATACGTGCTCCAAC
vha-6	AGAATGCGTACACGAGAAAA	ATGCTCATCGTAGTCTGGAA
vha-11	GGCAGATTTGGTGAAGGCTG	CGACGAGAGTGTCGCGTATT
Y16B4A.2	TGCACAAATGCTTCCACCCA	ATGCAGCTTGGTTTCTGTAG
ZK1307.8	GGAATGGAGTGGCCCAGAAG	ATTTTGCTGGCTCCGTGACT

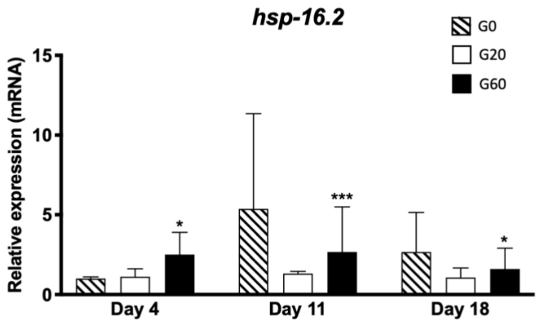

Figure S1: Relative expression of heat shock protein 16.2 (HSP-16.2) encoding gene. Young adult worms were fed for 4, 11 and 18 days with bacteria, which were pre-treated with 0 mM (G0), 20 mM (G20) or 60 mM (G60) of glyoxylic acid. Relative expression of genes coding for antioxidant, carbonyl stress and unfolded protein responses were analyzed by comparing all the conditions to the Ctrl at day 4. Data were expressed as means ± standard deviation of 3 biological replicates. The *cdc-42* and *pmp-3* genes were used to normalize levels of gene expression. *$p \leq 0.05$, ***$p \leq 0.001$ G20 or G60 *vs* G0 for the same incubation time (Kruskal−Wallis, Dunn's multiple comparisons test).

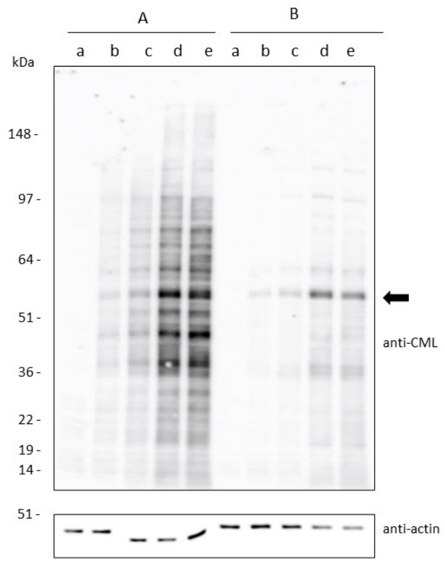

Figure S2: Digestion of bacterial glycated proteins by *C. elegans*. Young adult worms were fed with bacteria, which were incubated with 0 mM (G0) and 60 mM (G60) of glyoxylic acid. Some worms were only incubated with either G0 (a) or G60 (e) bacteria, while others were incubated with a mixed of the 2 bacteria types with a ratio G0:G60 of 7:1 (b), 3:1 (c) and 1:1 (d). After 4 days of culture, half of the worms were lysed (A) while the other half was incubated in the bacteria-free medium for 1 day before being washed and lysed (B). Western blots of worms' lysates were stained with anti-CML and anti-actin antibodies. The arrow shows a protein band poorly digested by the worms.

All results are representative of 3 independent experiments.

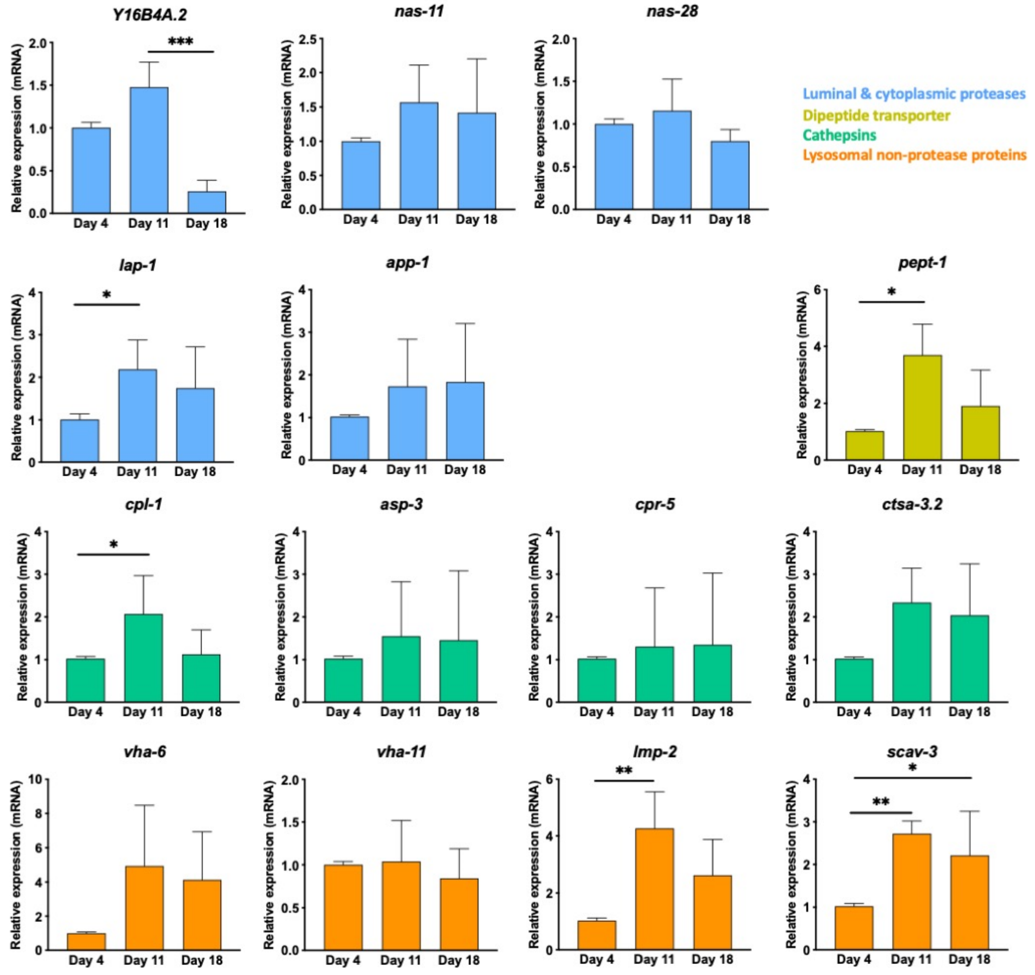

Figure S3: Impacts of dietary *Nε*-carboxymethyllysine (dCML) exposure on transcriptional regulation of some digestive enzymes and lysosomal proteins. Young adult worms were fed with bacteria, which were incubated with 0 mM of glyoxylic acid. After 4, 11, and 18 days of culture, relative expression of genes coding for luminal and/or cytoplasmic proteases, lysosomal cathepsins and lysosomal non-protease proteins were analyzed by comparing all the conditions to the day 4. Data were expressed as means ± standard deviation of 3 biological replicates. The *cdc-42* and *pmp-3* genes were used to normalize levels of gene expression.

*p≤0.05, **p≤0.01, ***p≤0.001 (Kruskal–Wallis, Dunn's multiple comparisons test).

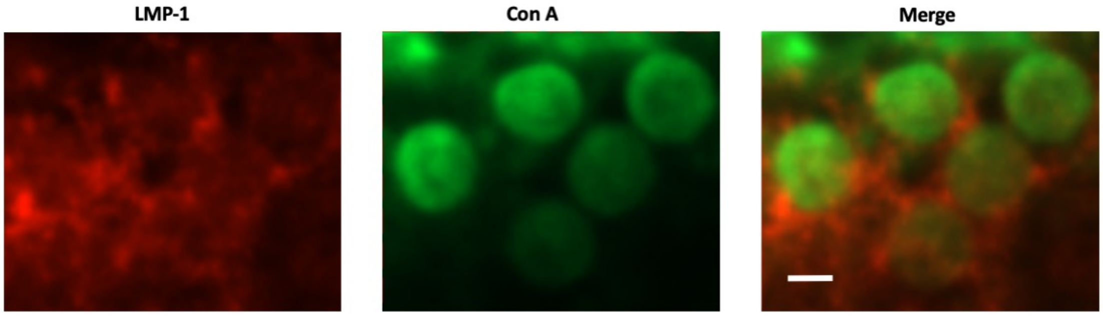

Figure S4: Mapping of lysosomal epitope and concanavalin A (Con A) staining on intestinal endosomes. Immunohistochemical assays were performed on worms fed for 4 days with bacteria, which were pretreated with 60 mM of glyoxylic acid, using Con A and and anti-lysosome-associated membrane protein homolog 1 (LMP-1) antibody. Fluorescence micrographs with the mentioned lectin or antibody are shown. Scale bars = 2.5 μm.

All results are representative of 3 independent experiments.
